# Metabolic syndrome and kidney dysfunction: emerging molecular and cellular mechanisms at the metabolic–renal interface

**DOI:** 10.3389/fendo.2026.1785937

**Published:** 2026-04-17

**Authors:** William R. Marshall, Darren Green, Smeeta Sinha, Philip A. Kalra

**Affiliations:** 1 Division of Cardiovascular Sciences, University of Manchester, Manchester, United Kingdom; 2 Donal O’Donoghue Renal Research Centre, Northern Care Alliance NHS Foundation Trust, Salford, United Kingdom

**Keywords:** albuminuria, chronic kidney disease, insulin resistance, lipotoxicity, metabolic syndrome, mitochondrial dysfunction

## Abstract

Metabolic syndrome and chronic kidney disease frequently coexist, acting synergistically to amplify the risk of adverse cardiovascular and renal outcomes. Large epidemiological studies now identify metabolic syndrome as an independent determinant of incident chronic kidney disease, accelerated estimated glomerular filtration rate decline and progression to end-stage kidney disease, even after adjustment for diabetes and hypertension. These observations have driven a conceptual shift away from haemodynamic and glomerular hyperfiltration-centric models towards an integrated paradigm of metabolic dysfunction–associated kidney disease. This framework emphasises systemic insulin resistance, lipotoxicity, chronic low-grade inflammation and disrupted cellular energy homeostasis as central drivers of renal injury. Within the kidney, metabolic syndrome promotes renal microvascular rarefaction, endothelial glycocalyx disruption, podocyte injury and tubular–interstitial inflammation. At the cellular and molecular level, key mechanisms include dysregulated adipokine and hepatokine signalling; ectopic lipid accumulation and renal lipotoxicity; mitochondrial dysfunction; endoplasmic reticulum stress; defective autophagy and mitophagy; oxidative stress and epigenetic remodelling. Importantly, several established therapies including renin–angiotensin–aldosterone system blockade, non-steroidal mineralocorticoid receptor antagonists and sodium–glucose cotransporter-2 inhibitors appear to confer renoprotection through modulation of these metabolic and cellular stress pathways. In this focused narrative review, we summarise the recent advances in the molecular and cellular mechanisms linking metabolic syndrome to kidney dysfunction. We highlight the key knowledge gaps and outline potential future therapeutic opportunities at the metabolic–renal interface.

## 1 Introduction

Metabolic syndrome (MetS), defined by central obesity, dysglycaemia, dyslipidaemia and hypertension, affects approximately 20–25% of adults worldwide and continues to rise in parallel with the global epidemics of obesity and type 2 diabetes (1). Beyond its established cardiovascular consequences, there is growing evidence that MetS is an important determinant of chronic kidney disease (CKD), accelerated estimated glomerular filtration rate (eGFR) decline and progression to kidney failure, independent of the presence of diabetes or hypertension (2–8).

These observations have driven growing recognition of MetS-associated kidney disease, in which renal injury arises from systemic insulin resistance, adiposity and dyslipidaemia rather than classical haemodynamic or hyperglycaemia-centric mechanisms alone. While earlier paradigms emphasise glomerular hyperfiltration, intrarenal renin–angiotensin–aldosterone system (RAAS) activation and hypertension, emerging experimental and clinical data now reframe MetS-associated kidney disease as a multi-dimensional disorder characterised by disordered lipid handling, mitochondrial dysfunction, chronic inflammatory signalling and maladaptive intracellular stress responses (9–13). In this review, MetS is considered across the continuum from insulin resistance and pre-diabetes to early type 2 diabetes, with emphasis on metabolic drivers of renal injury that are mechanistically distinct from, and not limited to, classical diabetic kidney disease.

Despite these advances, mechanistic insights have not yet been fully integrated into a coherent metabolic–renal disease framework that informs biomarker development, patient stratification or mechanism-targeted therapy. We synthesise recent epidemiological, experimental and translational evidence linking MetS to kidney dysfunction and highlight emerging therapeutic opportunities at the metabolic–renal interface.

**Figure 1:**
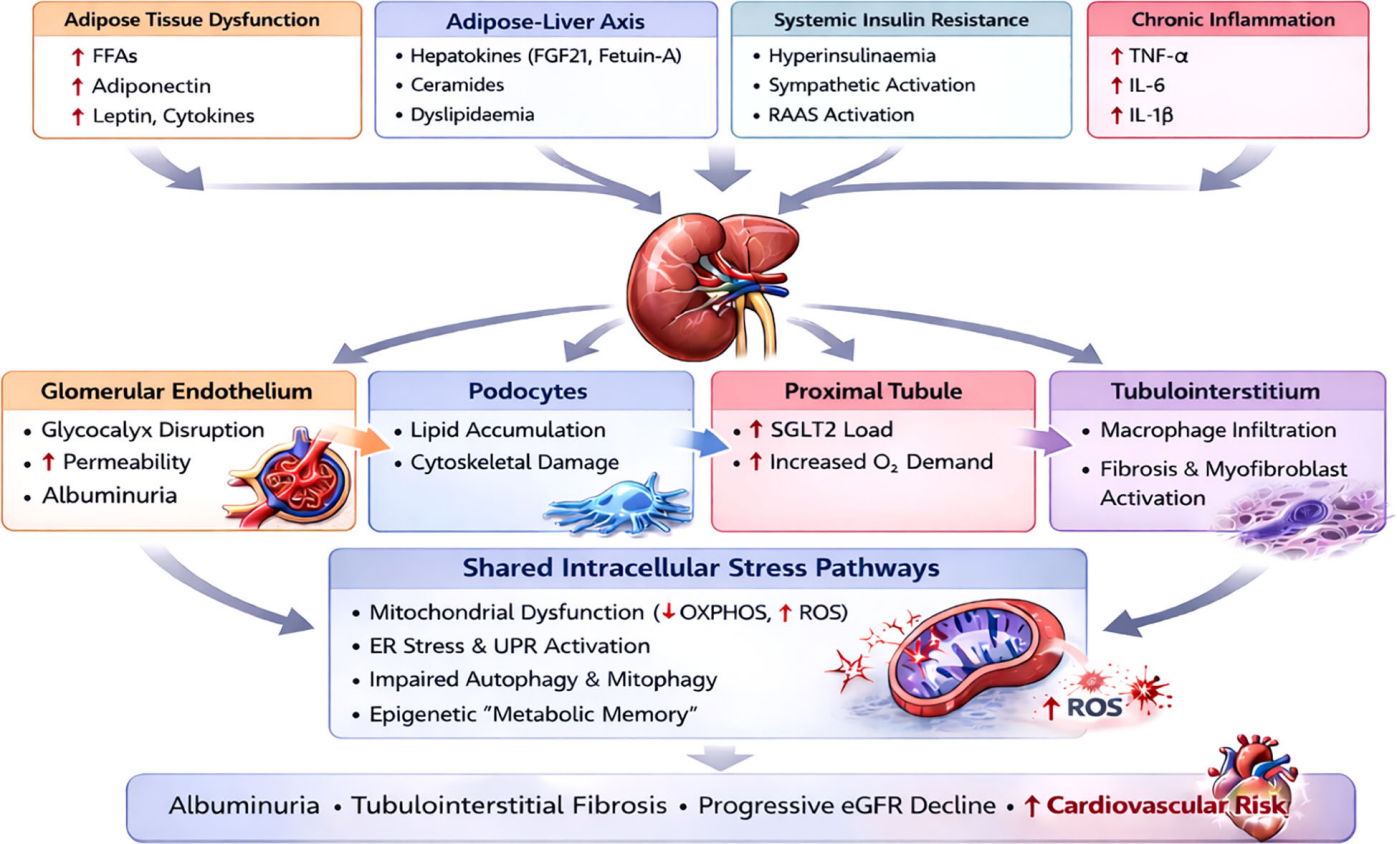
Systemic metabolic drivers, renal cell–specific injury and shared intracellular stress pathways in MetS-associated kidney disease. Diagram showing adipose tissue dysfunction, the adipose–liver axis, systemic insulin resistance and chronic inflammation acting as upstream drivers of renal injury. They differentially affect glomerular endothelial cells, podocytes, proximal tubular cells and the tubulointerstitium, collectively promoting albuminuria and fibrogenesis. Across renal cell types, common intracellular mechanisms include mitochondrial dysfunction, endoplasmic reticulum (ER) stress and unfolded protein response (UPR) activation, impaired autophagy/mitophagy and epigenetic “metabolic memory.” These convergent pathways drive albuminuria, tubulointerstitial fibrosis, progressive estimated glomerular filtration rate (eGFR) decline, and increased cardiovascular risk. FFAs, free fatty acids; FGF21, fibroblast growth factor 21; RAAS, renin-angiotensin-aldosterone system; TNF-α - tumour necrosis factor -α; IL-6 - interleukin-6; IL-1β - interleukin-1β; SGLT2 - sodium glucose co-transporter 2; OXPHOS, oxidative phosphorylation; ROS, reactive oxygen species; ER, endoplasmic reticulum; UPR, unfolded protein response.

The integrated metabolic–renal framework that underpins this review is summarised in **Figure 1**, which illustrates the interaction between systemic metabolic drivers, renal cell–specific injury and shared intracellular stress pathways.

## 2 Beyond diabetic kidney disease: clinical and epidemiological evidence for metabolic dysfunction-associated kidney disease

MetS and CKD frequently coexist, reflecting shared endocrine and metabolic disturbances including central adiposity, insulin resistance, dyslipidaemia, chronic low-grade inflammation and neurohormonal activation (14–16).

Large prospective cohort studies and meta-analyses have firmly established MetS as an independent risk factor for incident CKD, accelerated eGFR decline and progression to kidney failure (17, 18). A pooled meta-analysis of observational cohorts reported a ~50–70% increased risk of developing CKD among individuals with MetS, with consistent associations across geographic regions and ethnic groups (14, 19). Importantly, this excess renal risk persists after adjustment for diabetes and hypertension, indicating that kidney disease in MetS cannot be explained solely by hyperglycaemia or haemodynamic stress (4).

Studies using UK Biobank data further support a broader metabolic–renal disease continuum. Analyses demonstrate that individual MetS components (particularly central obesity, hypertriglyceridaemia and low high-density lipoprotein (HDL) cholesterol) are independently associated with future CKD risk, even in individuals without diagnosed diabetes or hypertension (4). Central obesity and insulin resistance appear to be especially potent drivers, closely linked to early albuminuria, glomerular hyperfiltration and subsequent eGFR decline (16). These observations underscore the importance of adipose tissue dysfunction, lipid flux and systemic insulin resistance as upstream determinants of renal injury.

MetS-associated kidney disease often presents with modest albuminuria and preserved or only mildly reduced eGFR in its early stages; yet, it is accompanied by a disproportionately high burden of cardiovascular disease (20, 21). This phenotype suggests that renal injury in MetS is initiated at the level of the microvasculature and metabolically active renal cell populations rather than through advanced nephron loss alone (21). Such features align with broader cardiometabolic disease patterns, in which early endothelial dysfunction and cellular metabolic stress precede overt structural organ damage.

The relationship between MetS and CKD is further amplified as kidney function declines. Progressive CKD exacerbates systemic insulin resistance, dyslipidaemia and inflammatory signalling through reduced insulin clearance, disordered adipokine and hepatokine profiles and chronic activation of neurohormonal and immune pathways (14, 16). These changes intensify metabolic stress across multiple organs, including the kidney itself, creating a vicious cycle that accelerates renal and cardiovascular disease progression.

Collectively, epidemiological evidence challenges models of CKD in MetS that focus predominantly on hyperglycaemia, blood pressure or glomerular haemodynamics. Instead, it supports an integrated endocrine framework in which chronic metabolic stress driven by insulin resistance, adipose tissue dysfunction, altered lipid handling and systemic inflammation targets vulnerable renal cell types, including endothelial cells, podocytes and proximal tubular epithelium. This perspective provides the rationale for examining the molecular and cellular mechanisms linking MetS to kidney dysfunction.

## 3 Systemic milieu of metabolic syndrome

This section focuses on systemic metabolic and inflammatory drivers that precede, amplify, or modulate classical haemodynamic kidney injury. Rather than revisiting well-established blood pressure-centric paradigms, we review upstream endocrine and metabolic abnormalities including insulin resistance, chronic low-grade inflammation, organ-derived lipotoxic signalling and metabolically driven neurohormonal activation. These are central to the canonical definition of MetS and exert direct effects on renal cellular homeostasis. Haemodynamic stress is incorporated as a downstream amplifier within this metabolic framework rather than treated as an isolated parallel mechanism.

### 3.1 Insulin resistance

Within non-diabetic, pre-diabetic and early diabetic states, insulin resistance is considered to be the dominant upstream abnormality; dysglycaemia and glucotoxicity are considered parallel or downstream stressors that increasingly contribute to renal injury as metabolic disease progresses. Insulin resistance is strongly associated with microalbuminuria and incident CKD (22–24). Mechanistically, experimental models demonstrate that systemic insulin resistance enhances adipose tissue lipolysis, leading to elevations in circulating free fatty acids (FFAs) that exceed renal lipid-handling and oxidative capacity (11).

Compensatory hyperinsulinaemia further amplifies renal injury by increasing sympathetic nervous system activity, promoting tubular sodium reabsorption and activating the RAAS, thereby contributing to glomerular hyperfiltration and hypertension (11). In parallel, insulin resistance drives metabolic inflexibility, characterised by impaired switching between glucose and fatty acid oxidation in renal tubular cells, resulting in mitochondrial substrate overload, excess reactive oxygen species (ROS) generation and tubular injury (11).

Glucotoxic mechanisms are acknowledged as important co-contributors across the MetS–diabetes continuum. Chronic hyperglycaemia promotes advanced glycation end-product (AGE) formation and AGE-RAGE signalling, inducing oxidative stress, endothelial dysfunction and inflammatory gene expression. These pathways interact bidirectionally with lipotoxic and mitochondrial stress, particularly in later disease stages. Given the extensive existing literature on established diabetic kidney disease, these mechanisms are referenced here without further detailed expansion.

Collectively, insulin resistance provides a mechanistic link between systemic adiposity and renal lipotoxicity, mitochondrial dysfunction and haemodynamic amplification in MetS, supporting its role as a therapeutic target upstream of glucose lowering alone.

### 3.2 Chronic inflammation

Chronic low-grade inflammation represents a central, integrative pathway linking insulin resistance, adipose tissue dysfunction, metabolic dysfunction-associated liver disease (MASLD) and CKD. Adipose tissue macrophage infiltration, hepatokine dysregulation and innate immune activation drive persistent elevations in inflammatory mediators, including tumour necrosis factor-α (TNF-α), interleukin-6 (IL-6) and interleukin-1β (IL-1β), which exert direct effects on renal endothelial cells, podocytes and tubular epithelium (25). Large human cohort studies including the chronic renal insufficiency cohort (CRIC) demonstrate strong independent associations between these circulating inflammatory biomarkers and CKD progression, albuminuria, cardiovascular events and mortality; this reinforces the relevance of inflammatory signalling in MetS-associated kidney disease (26, 27). In experimental models, inflammatory signalling not only amplifies metabolic stress but also interacts bidirectionally with mitochondrial dysfunction, endoplasmic reticulum (ER) stress and impaired autophagy, creating self-sustaining injury loops within the kidney (28).

Systemic inflammation and oxidative stress are tightly coupled processes in MetS. While this section focuses on the circulating inflammatory milieu and immune activation, downstream redox imbalance and ROS generation are discussed in detail later as intrinsic cell consequences of mitochondrial and metabolic dysfunction.

Collectively, these findings position chronic low-grade inflammation as both a mediator and amplifier of MetS-associated kidney disease, with circulating inflammatory biomarkers offering potential tools for risk stratification and therapeutic targeting.

### 3.3 Systemic organ crosstalk and lipotoxic signalling

Adipose tissue and liver dysfunction in MetS contribute to a shared endocrine and lipotoxic milieu characterised by excess FFAs, toxic lipid species and organ-derived mediators that converge on the kidney through overlapping inflammatory and metabolic pathways.

MASLD, which is highly prevalent in MetS, amplifies this systemic disturbance through increased circulating FFAs, ceramides and hepatokines, including fetuin-A and fibroblast growth factor 21 (FGF21) (29, 30). These factors exacerbate insulin resistance, endothelial dysfunction and renal lipid accumulation, embedding the kidney within a broader network of metabolic stress and organ crosstalk (31–34).

Dysregulated adipokine signalling further links adipose tissue dysfunction to kidney injury. Elevated leptin, resistin and pro-inflammatory cytokines, together with reduced adiponectin, have been consistently associated with higher CKD prevalence and more rapid disease progression (35–37). Mechanistic studies indicate that leptin promotes sympathetic activation, glomerular hypertrophy, extracellular matrix deposition and profibrotic transforming growth factor-β (TGF-β) signalling in mesangial and tubular cells. In contrast, adiponectin exerts anti-inflammatory and anti-fibrotic effects through activation of AMP-activated protein kinase (AMPK) and peroxisome proliferator-activated receptor-α (PPAR-α) pathways (38–40). Paradoxical elevations in circulating adiponectin observed in advanced CKD likely reflect impaired renal clearance and compensatory upregulation rather than preserved protective signalling (41).

FGF21 has emerged as a central endocrine mediator at the interface of hepatic, adipose and renal metabolism (42–44). Circulating concentrations increase in obesity, insulin resistance and CKD, consistent with a state of relative FGF21 resistance. Large population-based studies demonstrate independent associations between elevated FGF21 levels and adverse kidney outcomes, including accelerated eGFR decline and incident kidney failure, supporting translational relevance beyond experimental models (45, 46). In preclinical systems, FGF21 signalling enhances insulin sensitivity, promotes fatty acid oxidation, suppresses inflammatory pathways and attenuates lipotoxic injury in renal and vascular tissues (47, 48). Additional adipokines, including chemerin and visfatin, further modulate endothelial function, oxidative stress and RAAS activity, reinforcing the role of dysfunctional adipose tissue in driving renal inflammation and fibrosis (49–51).

Take-home: Adipose–liver–kidney crosstalk in MetS creates a lipotoxic and inflammatory endocrine milieu that embeds the kidney within a broader cardiometabolic disease network.

### 3.4 Metabolically driven hypertension and haemodynamic amplification

Hypertension in MetS is not a purely haemodynamic phenomenon but is tightly coupled to insulin resistance, adipokine dysregulation and neurohormonal activation (52). Hyperinsulinaemia promotes sympathetic nervous system activation and renal sodium retention, while adipokines such as leptin directly stimulate sympathetic outflow and augment RAAS signalling. These pathways increase salt sensitivity, intraglomerular pressure and renal oxygen demand (53).

In parallel, chronic metabolic inflammation and oxidative stress drive microvascular rarefaction and endothelial dysfunction, impairing renal autoregulation and increasing susceptibility to ischaemia (54, 55). Together, these mechanisms position hypertension as a downstream haemodynamic amplifier of metabolically initiated renal injury, linking systemic insulin resistance and adiposity to glomerular hyperfiltration, endothelial stress and progressive nephron loss (56).

Collectively, these systemic metabolic and inflammatory perturbations exert their pathogenic effects by targeting specific renal cell populations with distinct metabolic vulnerabilities, which are considered in the next section.

## 4 Renal cell types in systemic metabolic stress

### 4.1 Glomerular endothelial cells and glycocalyx integrity

Glomerular endothelial cells are an early and preferential target of metabolic stress in MetS-associated kidney disease, with injury to the luminal endothelial glycocalyx (EG) representing a structural correlate of endothelial dysfunction (57). The EG is a negatively charged meshwork of proteoglycans and glycosaminoglycans that governs vascular permeability, shear sensing and leukocyte adhesion.

Across experimental and clinical CKD cohorts, progressive EG degradation is observed with advancing disease stage, with circulating glycocalyx shed markers correlating with declining kidney function and albuminuria (58). Metabolic drivers of EG injury include uremic toxins, oxidative stress, hyperglycaemia and dyslipidaemia (59), which collectively increase endothelial permeability, promote leukocyte adhesion and microthrombus formation, and impair HDL function while favouring pro-atherogenic lipoprotein profiles (60–62). These processes further destabilise the glomerular filtration barrier and amplify albumin leakage (63–67). Inflammatory chemokine signalling, including endothelial C-X-C chemokine receptor 2 (CXCR2) activation, has been implicated in glycocalyx shedding and endothelial injury, highlighting a potential therapeutic target in MetS-related glomerular damage (68).

Together, these data define a convergent pathway in which systemic insulin resistance, chronic inflammation and haemodynamic stress drive endothelial activation and glycocalyx loss, leading to impaired shear sensing, microvascular dysfunction and albuminuria. These processes are reflected in circulating glycocalyx markers, endothelial inflammatory signatures and microvascular imaging abnormalities, and represent potentially modifiable targets, particularly through RAAS blockade and mineralocorticoid receptor antagonism, with emerging interest in glycocalyx-stabilising strategies.

Take-home: EG injury represents an early, potentially modifiable lesion linking metabolic stress to albuminuria and microvascular dysfunction in MetS–associated kidney disease.

### 4.2 Podocytes: cytoskeletal stress, lipotoxicity and autophagy

Podocytes are highly specialised, terminally differentiated cells that are vulnerable to metabolic and mechanical stress. In obesity and diabetes, converging evidence implicates ectopic lipid accumulation, mitochondrial dysfunction, oxidative stress and defective autophagy as central drivers of podocyte injury (69, 70). Increased FFA flux, impaired mitochondrial β-oxidation and disordered lipid droplet handling promote intracellular lipotoxicity, rendering podocytes susceptible to injury.

Specific lipid intermediates appear to mediate these effects. Ceramides accumulate in podocytes exposed to saturated fatty acids and have been shown to promote mitochondrial outer membrane permeabilisation, ROS production and activation of pro-apoptotic pathways (51). Diacylglycerols (DAGs) may activate protein kinase C isoforms, further amplifying oxidative stress (71). Long-chain and very-long-chain ceramide species have been associated with CKD progression in human cohort studies, supporting translational relevance (72). These bioactive lipids therefore represent plausible mechanistic mediators linking systemic dyslipidaemia to glomerular injury.

These metabolic insults disrupt actin cytoskeletal organisation and slit diaphragm integrity through ROS generation, AGE signalling and angiotensin II activation, culminating in foot process effacement and proteinuria (73).

In parallel, impaired autophagy and lysosomal clearance leads to accumulation of damaged mitochondria and misfolded proteins, further amplifying cellular stress (74). Activation of inflammatory pathways including nuclear factor kappa-light-chain-enhancer of activated B cells (NF-κB), nucleotide-binding oligomerisation domain (NOD)-like receptors, and the NOD-like receptor family pyrin domain containing 3 (NLRP3) inflammasome drive local release of IL-1β and interleukin-18 (IL-18), promoting macrophage recruitment and sustained glomerular injury (75).

Collectively, podocyte injury in MetS reflects a convergent axis in which systemic lipotoxicity, inflammatory cytokines and RAAS activation drive mitochondrial dysfunction, oxidative stress and cytoskeletal instability. Failure of compensatory autophagy further amplifies structural injury to the glomerular filtration barrier, culminating in proteinuria. These processes are increasingly reflected in emerging transcriptomic and proteomic signatures of oxidative stress, disordered lipid handling and cytoskeletal remodelling, and together highlight potential therapeutic opportunities targeting lipotoxic flux, mitochondrial homeostasis and RAAS signalling.

Take-home: Podocyte injury in MetS reflects convergence of lipotoxicity, mitochondrial dysfunction and defective autophagy, leading to cytoskeletal instability and proteinuria.

### 4.3 Mesangial cells: metabolic stress and glomerular remodelling

Mesangial cells represent a central component of the glomerular filtration unit and play a key role in adaptive and maladaptive responses to metabolic and haemodynamic stress. In MetS, mesangial cells are exposed to insulin resistance, lipotoxic mediators and angiotensin II, promoting extracellular matrix deposition, inflammatory cytokine release and heightened sensitivity to mechanical stretch (76–79). These responses contribute to mesangial expansion, altered capillary architecture and progression toward glomerulosclerosis, completing the triad of endothelial, podocyte and mesangial injury in MetS-associated kidney disease (77).

Take-home: Mesangial cells integrate metabolic and haemodynamic stress signals, driving maladaptive extracellular matrix remodelling that amplifies glomerular injury in MetS.

### 4.4 Proximal tubule: mitochondrial overload and AKT serine/threonine kinase 1 signalling

The proximal tubule (PT) is a metabolically demanding nephron segment reliant on mitochondrial oxidative phosphorylation. In MetS, increased sodium–glucose reabsorption via SGLT2 elevates adenosine triphosphate (ATP) demand and oxygen consumption, while heightened exposure to luminal and peritubular glucose, FFAs and uric acid promotes mitochondrial ROS generation, lipid accumulation and deoxyribonucleic acid (DNA) damage (80, 81).

Recent experimental work identifies mitochondrial AKT serine/threonine kinase 1 [AKT1 - also known as protein kinase B (PKB)] signalling as a key integrator of metabolic stress in PT cells. Activation of this pathway contributes to mitochondrial dysfunction, tubular injury and albuminuria in MetS models, whereas genetic or pharmacological modulation ameliorates injury (80). In parallel, renal lipotoxicity induces ER stress, apoptosis and necroptosis, alongside metabolic reprogramming away from fatty acid oxidation toward glycolysis and glutaminolysis, mediated by peroxisome proliferator-activated receptor gamma coactivator-1 alpha (PGC-1α), hypoxia-inducible factor-1 alpha (HIF-1α) and AMP-activated protein kinase (AMPK) signalling (82–85). This shift reduces ATP efficiency and exacerbates tubular hypoxia.

Importantly, these metabolic disturbances occur within a microvascular environment characterised by capillary rarefaction and impaired oxygen delivery. In the setting of increased ATP demand and oxygen consumption, capillary rarefaction and impaired oxygen delivery render the PT highly susceptible to relative hypoxia. Stabilisation of HIF-1α under these conditions promotes glycolytic reprogramming, inflammatory signalling and profibrotic gene expression, positioning hypoxia as a central amplifier linking metabolic overload to tubulointerstitial fibrosis.

In tubular epithelial cells, saturated fatty acids, ceramides and toxic acylcarnitine species accumulate when fatty acid oxidation capacity is exceeded. Ceramide-driven mitochondrial dysfunction promotes ferroptosis and apoptosis, while DAG-mediated protein kinase C (PKC) activation enhances inflammatory and profibrotic signalling (51). Unlike podocytes, tubular cells exhibit marked lipid droplet accumulation and transcriptional suppression of fatty acid oxidation genes, creating a self-reinforcing cycle of lipid retention, oxidative stress and interstitial fibrosis.

Given the PT’s disproportionate reliance on mitochondrial fatty acid oxidation, even modest suppression of fatty acid oxidation results in rapid lipid accumulation and energetic stress, predisposing to fibrotic remodelling. In contrast, lipid-induced oxidative stress in podocytes primarily disrupts cytoskeletal integrity and filtration barrier function.

PT injury in MetS reflects a coordinated response to substrate overload and impaired metabolic flexibility, with mitochondrial dysfunction, oxidative stress and defective autophagy driving tubular damage and fibrotic remodelling. These processes are increasingly reflected in tubular transcriptomic and metabolomic signatures of impaired fatty acid oxidation and oxidative stress, and highlight potential therapeutic opportunities, including SGLT2 inhibition, AMPK-modulating approaches and emerging mitochondrial-directed strategies.

Take-home: The PT is a key metabolic “stress sensor” in MetS, where substrate overload and impaired mitochondrial flexibility drive tubular injury and fibrotic remodelling.

### 4.5 Tubulointerstitial compartment: fibroblasts, pericytes and immune infiltration

Human biopsy studies and cohort analyses demonstrate that MetS is associated with chronic tubulointerstitial inflammation characterised by macrophage accumulation, T-cell activation and sustained cytokine production (87, 88). Crosstalk between injured tubular epithelium, resident fibroblasts, pericytes and infiltrating immune cells promotes pericyte-to-myofibroblast transition and extracellular matrix expansion via TGF-β, connective tissue growth factor (CTGF) and platelet-derived growth factor (PDGF) signalling pathways (89).

Experimental data further suggest that epigenetic reprogramming including DNA methylation, histone modification and non-coding ribonucleic acid (RNA) regulation locks tubular and stromal cells into a persistent pro-inflammatory and profibrotic state (90–92). This loop sustains fibroblast activation, capillary rarefaction and progressive interstitial fibrosis.

Overall, the tubulointerstitial response in MetS reflects a dominant axis linking systemic metabolic inflammation to immune recruitment, stromal activation and progressive extracellular matrix accumulation. These processes are reflected in fibrotic and inflammatory transcriptomic signatures, increasing interstitial macrophage burden and declining peritubular capillary density, and are strongly associated with subsequent kidney function decline in MetS.

Take-home: Tubulointerstitial inflammation and fibroblast activation represent final common pathways linking systemic metabolic stress to irreversible kidney function loss.

### 4.6 Convergence of cell-specific injury

While these cell-specific injuries appear heterogeneous at the structural level, they converge on a shared set of intracellular stress responses that ultimately determine cell survival, inflammation and fibrotic remodelling. These are discussed in the next section.

## 5 Intracellular stress pathways

### 5.1 Mitochondrial dysfunction and metabolic reprogramming

Recent reviews and experimental studies converge on mitochondrial dysfunction as a central pathogenic hub linking MetS to kidney injury across multiple renal cell types (93–95). Hallmark features include fragmentation of the mitochondrial network driven by excessive dynamin-related protein-1 (DRP1) mediated fission; loss of mitofusin-dependent fusion; and impaired mitochondrial biogenesis due to suppression of PGC-1α and mitochondrial transcription factor A (TFAM) (96, 97). These structural abnormalities are accompanied by reduced oxidative phosphorylation capacity and ATP generation, with a compensatory shift toward glycolytic metabolism in tubular and glomerular cells (98).

Accumulation of damaged mitochondria and mitochondrial DNA further promotes renal injury by acting as damage-associated molecular patterns (DAMPs) that activate innate immune and inflammatory signalling pathways (99). Concomitantly, increased mitochondrial ROS production drives lipid peroxidation, DNA damage, and activation of redox-sensitive transcription factors; these include nuclear factor-κB (NF-κB) and activator protein-1 (AP-1) (100–102).

In humans, bulk and single-cell transcriptomic analyses of kidney biopsy tissue in MetS-associated kidney disease reveal cell-type-specific metabolic signatures, demonstrating distinct patterns of mitochondrial reprogramming across podocytes, proximal tubular cells, endothelial cells, and infiltrating immune populations (103).

Mitochondrial vulnerability in MetS is not uniform across renal cell types. Proximal tubular epithelial cells rely predominantly on mitochondrial fatty acid β-oxidation to meet high ATP demand, rendering them particularly vulnerable to fatty acid oxidation suppression and energetic stress when PGC-1α and PPAR-α signalling are impaired (104, 105). In contrast, podocytes display greater metabolic flexibility but are highly sensitive to lipid-induced mitochondrial ROS generation and cytoskeletal perturbation (106, 107). These intrinsic differences in substrate dependence likely contribute to the characteristic pattern of progressive tubular atrophy and interstitial fibrosis observed in metabolic kidney disease, whereas podocyte injury more prominently manifests as proteinuria.

Take-home: Mitochondrial dysfunction represents a central intracellular hub through which diverse metabolic insults converge to drive renal inflammation, fibrosis and functional decline.

### 5.2 Endoplasmic reticulum stress and unfolded protein response

In experimental models, glucolipotoxic stress activates the unfolded protein response (UPR) through the protein kinase R-like endoplasmic reticulum kinase (PERK), inositol-requiring enzyme 1 (IRE1) and activating transcription factor 6 (ATF6) signalling branches (108). When persistent, this ER stress response shifts from adaptive to maladaptive, promoting inflammatory signalling and apoptosis. In proximal tubular cells, saturated fatty acids and toxic lipid intermediates intensify C/EBP Homologous Protein (CHOP)-mediated apoptosis and synergise with mitochondrial dysfunction (109). By contrast, in podocytes, UPR activation destabilises slit diaphragm proteins and cytoskeletal architecture, while amplifying TGF-β and angiotensin II signalling, thereby exacerbating proteinuria (110).

### 5.3 Autophagy, mitophagy and lysosomal pathways

Autophagy and mitophagy represent essential mitochondrial quality-control mechanisms that are activated in response to metabolic and oxidative stress; failure of these adaptive pathways links mitochondrial dysfunction to sustained inflammation, fibrosis and cell death (110).

In metabolic overload, selective autophagy pathways are of particular significance. Lipophagy facilitates clearance of excess lipid droplets and limits lipotoxic injury, while ER-phagy mitigates sustained UPR activation under glucolipotoxic conditions (111). Disruption of these pathways promotes lipid accumulation, mitochondrial dysfunction and inflammatory signalling. Importantly, autophagy demonstrates context-dependent effects: inadequate activation predisposes to cellular injury, whereas persistent or dysregulated activation may contribute to tubular atrophy, underscoring its dual role in disease progression.

Human biopsy and transcriptomic studies in MetS-associated kidney disease demonstrate suppressed autophagic flux and impaired lysosomal function in both podocytes and tubular cells (112, 113). Accumulation of dysfunctional mitochondria and protein aggregates reflects failure of this adaptive quality-control system. Conversely, genetic or pharmacologic enhancement of autophagy including AMPK activation and SGLT2 inhibition attenuates experimental renal injury (114, 115). This suggests that autophagy represents a compensatory mechanism that becomes insufficient in the sustained stress of MetS.

### 5.4 Regulated cell death

Downstream of mitochondrial dysfunction and ER stress, regulated cell death pathways directly contribute to nephron loss in MetS-associated kidney disease. Intrinsic apoptosis is triggered by mitochondrial ROS accumulation, cytochrome-c release and caspase activation (93). In addition, ferroptosis, a regulated iron-dependent form of cell death driven by lipid peroxidation, has emerged as a pathway linking lipotoxicity, oxidative stress and tubular injury (116, 117). The lipid-rich and oxidative microenvironment characteristic of MetS creates conditions that favour ferroptotic cell death within the kidney.

### 5.5 Epigenetic reprogramming

Persistent metabolic stress and inflammation induce epigenetic changes in human cohorts (large epigenome-wide association studies) including DNA methylation, histone modifications and non-coding RNAs that sustain pathogenic transcriptional programs even after partial risk-factor control (118, 119). These “metabolic memories” may explain why renal risk remains high despite improvements in glycaemia or blood pressure, and underscore the need for early and comprehensive metabolic intervention.

### 5.6 Convergence of intracellular stress pathways

These intracellular stress pathways do not operate in isolation, but instead form an interconnected network. Mitochondrial dysfunction increases ROS generation, which exacerbates ER stress and impairs autophagic flux. Defective autophagy permits accumulation of damaged mitochondria, further amplifying oxidative stress and inflammatory signalling. Lipotoxic lipid species promote both ferroptotic vulnerability and epigenetic reprogramming, while hypoxia-driven metabolic shifts reinforce mitochondrial fragmentation and fibrotic gene expression. Collectively, this self-sustaining network shifts transient metabolic stress into persistent cellular injury and maladaptive remodelling.

Importantly, these convergent pathways may represent potentially modifiable targets that intersect with established and emerging therapies, as discussed below.

## 6 Therapeutic implications and emerging targets

**Figure 2:**
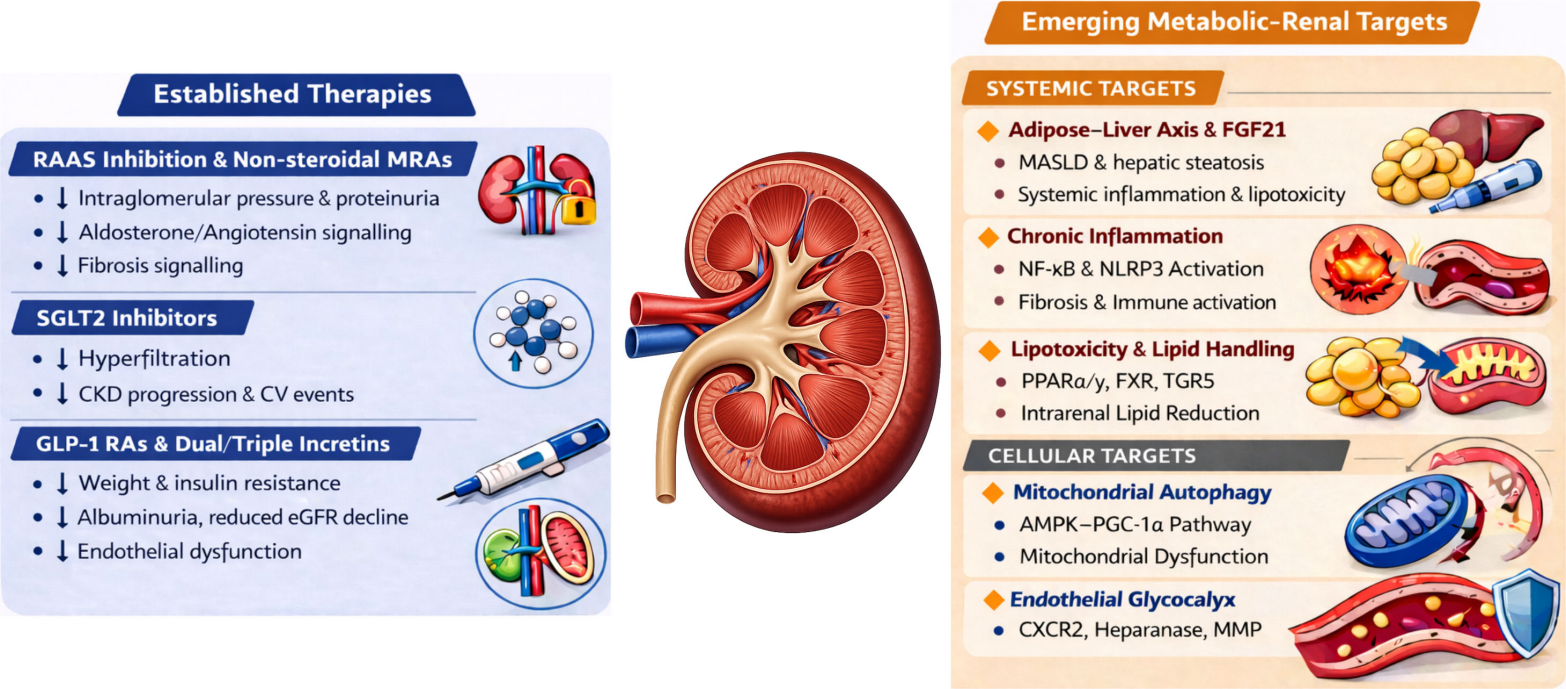
Established therapies and emerging targets in MetS-associated kidney disease. Left panel shows established therapies with robust human outcome data including renin–angiotensin–aldosterone system (RAAS) inhibition, non-steroidal mineralocorticoid receptor antagonists (nsMRAs), sodium–glucose cotransporter-2 inhibitors (SGLT2 inhibitors) and glucagon-like peptide-1 receptor agonists (GLP-1 RAs). While haemodynamic benefits are supported by clinical trials, several proposed cellular and anti-inflammatory mechanisms remain incompletely validated in human kidney tissue. Right panel shows emerging therapeutic strategies aim to modulate upstream systemic and resulting cellular drivers of metabolic–associated kidney disease. Systemic targets include the adipose–liver axis, chronic inflammation and intrarenal lipotoxicity. Cellular targets include restoration of mitochondrial quality control and preservation of endothelial glycocalyx integrity. CKD, chronic kidney disease; CV, cardiovascular; eGFR, estimated glomerular filtration rate; FGF21, fibroblast growth factor 21; MASLD, metabolic-dysfunction associated steatotic liver disease; NF-κB, nuclear factor kappa-light-chain-enhancer of activated B cells; NLRP3, NOD-like receptor family pyrin domain containing 3; PPAR-α, peroxisome proliferator-activated receptor- α; PPAR-γ, peroxisome proliferator-activated receptor- γ; FXR, farnesoid X receptor; TGR5, Takeda G protein-coupled receptor 5; AMPK-PGC-1α, AMP-activated protein kinase–peroxisome proliferator-activated receptor gamma coactivator-1 α; CXCR2, C-X-C chemokine receptor 2; MMP, matrix metalloproteinase.

The relationship between established therapies and emerging metabolic–renal targets is summarised in **Figure 2**.

### 6.1 Existing therapies and the need for mechanistic insights

The antiproteinuric and renoprotective effects of RAAS inhibition and non-steroidal mineralocorticoid receptor antagonists (nsMRAs) are supported by robust human randomised trial data across high-risk MetS-CKD populations (120–122). In contrast, mechanistic effects beyond haemodynamic control such as attenuation of oxidative stress, inflammation, fibrosis and improvements in mitochondrial function are predominantly supported by experimental models and *in vitro* studies, with limited direct validation in human kidney tissue (123, 124).

The clinical efficacy of SGLT2 inhibitors in reducing CKD progression and cardiovascular events is supported by multiple large-scale human outcome trials in both diabetic and non-diabetic CKD (125, 126). Restoration of tubuloglomerular feedback, reductions in intraglomerular pressure and hyperfiltration, and haemodynamic effects are supported by human physiological studies. However, proposed cellular mechanisms including improvements in mitochondrial efficiency, enhanced autophagy and mitophagy, and suppression of inflammatory and fibrotic signalling are largely derived from preclinical models. Corroboration from human biopsy, transcriptomic and metabolomic data is emerging but still somewhat limited (127–129).

Weight loss, improved insulin sensitivity and cardiovascular risk reduction with glucagon-like-peptide 1 receptor agonists (GLP-1 RAs) and dual/triple incretin therapies are well established in human trials. Signals for renal benefit, including reductions in albuminuria and slower eGFR decline, are supported by secondary analyses of cardiovascular outcome trials and early kidney-focused studies (130, 131). However, direct kidney-specific mechanisms such as anti-inflammatory, natriuretic, endothelial-protective or mitochondrial effects remain largely inferred from experimental models. Data from human kidney tissue remain limited, highlighting a key translational gap despite strong clinical outcome signals.

### 6.2 Targeting lipotoxicity and lipid handling

Associations between dyslipidaemia, ectopic lipid accumulation and CKD risk are supported by human observational and genetic studies (132). However, therapeutic modulation of renal lipotoxicity via PPARα/γ, farnesoid X receptor (FXR) or Takeda G protein-coupled receptor 5 (TGR5) pathways, and reductions in toxic lipid species such as ceramides, are supported predominantly by preclinical evidence (51). Human data demonstrating direct renal lipid flux modulation or reversal of intrarenal lipotoxic injury remain sparse.

### 6.3 Mitochondrial and autophagy targeted therapies

Evidence linking mitochondrial dysfunction to CKD progression is supported by human omics studies showing altered metabolic and mitochondrial gene signatures (85, 89). Nonetheless, therapeutic strategies aimed at modulating mitochondrial dynamics, reducing mitochondrial ROS, or enhancing mitophagy and biogenesis (e.g. AMPK–PGC-1α activation) are currently supported almost exclusively by experimental models, with minimal direct interventional human evidence (86, 99).

### 6.4 Endothelial and glycocalyx protection

Human observational and physiological studies demonstrate glycocalyx degradation in diabetes, hypertension and CKD, and associations with albuminuria and microvascular dysfunction (60). However, targeted interventions to stabilise or restore the glycocalyx such as inhibition of heparanase, matrix metalloproteinases (MMP) or chemokine pathways (e.g. CXCR2) remain largely experimental, with evidence derived primarily from preclinical diabetic kidney disease models (133–135).

### 6.5 Adipose and liver directed therapies

Weight loss, exercise, bariatric surgery and incretin-based therapies have strong human evidence for improving cardiometabolic risk profiles and reducing albuminuria (126, 127, 136). However, the concept that adipose tissue remodelling or treatment of MASLD confers kidney protection via reductions in inflammatory and lipotoxic signalling remains largely inferential, supported mainly by mechanistic and observational studies rather than kidney-specific interventional trials (137, 138).

Clinically, FGF21 analogues have demonstrated favourable effects on hepatic steatosis, inflammation and fibrosis in MASLD trials, with accompanying improvements in systemic metabolic profiles (139–141). Although kidney outcomes have not been primary endpoints, the close mechanistic overlap between MASLD, systemic inflammation and MetS-associated kidney disease positions FGF21-based therapies as promising candidates for targeting shared metabolic–renal injury pathways. Dedicated renal mechanistic and outcome studies are now needed to define whether modulation of FGF21 signalling can directly attenuate kidney disease progression.

### 6.6 Chronic inflammation

The clinical relevance of inflammation in MetS is supported by cardiovascular outcome trials targeting inflammatory pathways. These demonstrate reductions in cardiovascular events alongside decreases in high-sensitivity C-reactive protein (hsCRP) independent of lipid or glycaemic lowering (142). Although robust kidney-specific outcome data are not yet available, these findings reinforce the concept that inflammation is a modifiable axis within metabolic dysfunction. However, whether systemic anti-inflammatory strategies modify renal cellular stress pathways or albuminuria trajectories independent of cardiovascular effects remains uncertain.

## 7 Knowledge gaps and future directions

While SGLT2 inhibitors and GLP-1 RAs have transformed cardiorenal risk reduction, a clear unmet need remains. Robust stratification tools are required to identify which patients derive maximal benefit and to guide pathway-specific therapies targeting mitochondrial energetics, adipokine/hepatokine signalling and endothelial integrity. In this context, FGF21 has emerged as a compelling candidate. Positioned at the intersection of adipose, hepatic and renal metabolism, FGF21 integrates systemic metabolic signalling with intracellular stress responses. Experimental data demonstrate favourable effects on insulin sensitivity, lipid oxidation, mitochondrial function, autophagy and inflammatory signalling.

CKD is characterised by elevated circulating FGF21 levels, suggesting a state of relative FGF21 resistance that may be amenable to pharmacological intervention. Interpretation of circulating FGF21 levels in CKD however warrants caution. Elevated concentrations may reflect reduced renal clearance, compensatory upregulation or acquired FGF21 resistance rather than direct pathogenic signalling. Levels are influenced by eGFR, systemic inflammation and nutritional status, complicating its use as a standalone biomarker.

Clinically, long-acting FGF21 analogues have shown promising effects on hepatic steatosis, inflammation and fibrosis in MASLD trials, alongside improvements in systemic metabolic and inflammatory profiles (**Table 1**). Despite mechanistic plausibility, kidney outcomes have not been systematically examined in these studies, and direct renal mechanistic data in humans are lacking. Dedicated trials are needed to determine whether FGF21 modulation directly attenuates renal lipotoxicity and cellular stress, and whether it provides clinical benefit alongside established cardiorenal therapies.

**Table 1:** Current clinical trial evidence for Fibroblast growth factor 21 analogues.

Agent	Trial phase	Study population	Primary endpoints	Outcomes
Pegbelfermin (BMS-986036)	Phase 2	Obesity; NASH/MASLD	Liver fat fraction (MRI-PDFF)	↓ Hepatic steatosis ↓ ALT/AST ↑ Adiponectin Improved lipid profile
Efruxifermin (AKR-001)	Phase 2 (Phase 3 ongoing)	NASH with fibrosis (F1–F3)	Liver histology -Fibrosis improvement	↓ Steatosis ↓ fibrosis stage ↑ insulin sensitivity ↓ triglycerides
Pegozafermin (BIO89-100)	Phase 2	NASH/MASLD	Liver fat reduction	↓ Liver fat ↓ atherogenic lipids ↑ adiponectin
LY2405319	Phase 2	Obesity; type 2 diabetes	Safety; Metabolic endpoints	↓ Weight ↓ Triglycerides ↑ Adiponectin Improved insulin sensitivity
BFKB8488A	Phase 1	Obesity; insulin resistance	Metabolic safety and efficacy	Improved lipid profile Modest glycaemic effects
MK-3655 (NGM313)	Phase 2	Obesity; insulin resistance	Insulin sensitivity	Improved insulin sensitivity

Table shows trials to date evaluating fibroblast growth factor (FGF21)-based therapies across obesity, metabolic dysfunction-associated steatotic liver disease (MASLD; previously non-alcoholic steatohepatitis (NASH)) and insulin-resistant populations. Reported primary endpoints largely reflect hepatic fat reduction (typically assessed by magnetic resonance imaging–proton density fat fraction (MRI-PDFF)), histological fibrosis improvement, or metabolic safety and efficacy measures. Across trials, consistent effects include reductions in hepatic steatosis and triglycerides, improvements in insulin sensitivity and adiponectin levels, and favourable modulation of atherogenic lipid profiles. While renal endpoints were not primary outcomes in these studies, the observed metabolic and anti-inflammatory effects provide mechanistic rationale for future evaluation of FGF21 analogues on renal outcomes.

MASLD, metabolic dysfunction-associated steatotic liver disease; NASH, non-alcoholic steatohepatitis; ALT, alanine aminotransferase; AST, aspartate aminotransferase.

Growing evidence supports chronic inflammation as a modifiable driver of cardio–renal risk in CKD. Dedicated inhibition of upstream inflammatory pathways, including IL-6 signalling, has emerged as a promising approach. Ongoing and recent trials of IL-6 pathway inhibition, such as ZEUS, have renewed interest in targeting inflammation upstream of atherosclerosis and organ injury (143–145). Whether IL-6 inhibition can directly modify kidney disease progression, albuminuria trajectories, or metabolic–renal phenotypes remains an important unanswered question.

The Study of Heart and Renal Protection (SHARP) trial demonstrated that intensive low-density lipoprotein (LDL)-cholesterol lowering with simvastatin–ezetimibe significantly reduced major atherosclerotic cardiovascular events in CKD, yet did not meaningfully alter CKD progression (146). This apparent dissociation suggests that renal lipotoxicity in MetS-associated kidney disease may not be determined solely by circulating lipoprotein burden, but instead by maladaptive intracellular lipid handling and lipid signalling pathways that are not addressed by conventional lipid-lowering therapies.

Addressing these challenges will require large, deeply phenotyped, multi-ethnic cohorts integrating metabolomics, lipidomics, proteomics and transcriptomics with longitudinal kidney outcomes. In parallel, interventional studies should incorporate kidney-specific endpoints alongside integrated biomarker assessment. Such approaches are necessary to resolve causal pathways, define metabolically driven kidney disease subtypes, and enable the development of mechanism-based precision therapies. Within this framework, FGF21 analogues represent a promising next-generation strategy to target shared metabolic–renal injury pathways and merit evaluation in future translational and clinical studies.

## 8 Conclusion

MetS-associated kidney disease is no longer adequately explained by glomerular hyperfiltration or blood pressure alone. Contemporary evidence paints a more complex picture in which insulin resistance, adipose and hepatic dysfunction, renal lipotoxicity, mitochondrial dysfunction and impaired quality control, endothelial glycocalyx disruption and chronic inflammation converge on vulnerable glomerular, tubular and microvascular cells. These insights reposition the kidney as a metabolically active, signalling organ within a wider network of systemic metabolic dysfunction.

Existing therapies, including RAAS inhibitors, SGLT2 inhibitors and nsMRAs likely derive substantial benefit through modulation of these interconnected molecular pathways. Therapies targeting lipotoxic flux, mitochondrial homeostasis, autophagy and endothelial integrity are now being actively explored.

Translating this expanding molecular understanding into clinical practice will require improved biomarkers, refined risk stratification and trials explicitly designed to test pathway-specific therapies. Integrating FGF21-based interventions with established cardiorenal protective agents represents a promising future direction, with the potential to address shared metabolic drivers of kidney, cardiovascular and liver disease.
